# Hygromycin B and Apramycin Antibiotic Resistance Cassettes for Use in *Campylobacter jejuni*

**DOI:** 10.1371/journal.pone.0095084

**Published:** 2014-04-21

**Authors:** Andrew Cameron, Erin C. Gaynor

**Affiliations:** Department of Microbiology and Immunology, University of British Columbia, Vancouver, British Columbia, Canada; Iowa State University, United States of America

## Abstract

*Campylobacter jejuni* genetic manipulation is restricted by the limited number of antibiotic resistance cassettes available for use in this diarrheal pathogen. In this study, two antibiotic resistance cassettes were developed, encoding for hygromycin B and apramycin resistance, for use in mutagenesis or for selection of gene expression and complementation constructs in *C. jejuni*. First, the marker genes were successfully modified to allow for insertional mutagenesis or deletion of a gene-of-interest, and were bracketed with restriction sites for the facilitation of site-specific cloning. These hygromycin B and apramycin markers are encoded by plasmids pAC1H and pAC1A, respectively. We also modified an insertional gene-delivery vector to create pRRH and pRRA, containing the hygromycin B and apramycin resistance genes, and 3 unique restriction sites for the directional introduction of genes into the conserved multi-copy rRNA gene clusters of the *C. jejuni* chromosome. We determined the effective antibiotic concentrations required for selection, and established that no harmful effects or fitness costs were associated with carrying hygromycin B or apramycin resistance under standard *C. jejuni* laboratory conditions. Using these markers, the arylsulfatase reporter gene *astA* was deleted, and the ability to genetically complement the *astA* deletion using pRRH and pRRA for *astA* gene insertion was demonstrated. Furthermore, the relative levels of expression from the endogenous *astA* promoter were compared to that of polycistronic mRNA expression from the constitutive promoter upstream of the resistance gene. The development of additional antibiotic resistance cassettes for use in *Campylobacter* will enable multiple gene deletion and expression combinations as well as more in-depth study of multi-gene systems important for the survival and pathogenesis of this important bacterium.

## Introduction

The relative paucity of genetic techniques available for the manipulation of *Campylobacter jejuni* has historically been a limiting factor in the study and molecular biology of the leading cause of bacterial gastroenteritis in the developed world [1]. *C. jejuni* is a member of a large genus of microaerophilic Gram-negative ε-proteobacteria and is carried harmlessly by many animals, especially poultry, but is an endemic cause of a range of diarrheal illnesses and medical complications in humans. Many laboratories are actively studying the bacterium to understand the genetic determinants and physiological features that contribute to *C. jejuni’s* virulence and prevalence as a food-borne enteric pathogen. Today, research in the area continues to benefit from and depends on a small arsenal of molecular tools, such as gene deletion strategies and plasmids for genetic complementation. Since the 1980’s, only selection for kanamycin and chloramphenicol resistance has been widely adopted for the genetic manipulation of *Campylobacter*.

The development of the first genetic tools for *C. jejuni* was precipitated after the demonstration of gene transfer from *Escherichia coli* to *C. jejuni* via plasmids carrying kanamycin resistance in 1987 [2]. This led to the development, in 1988, of a kanamycin resistance cassette for use in gene disruption experiments [3]. Cloning and expression of a chloramphenicol resistance gene from *Campylobacter coli* in 1990 [4] was followed by development of replicative cloning vectors and mutational constructs marked with chloramphenicol resistance in 1993 [5]. Approximately a decade later, three groups successfully mutagenized *C. jejuni* with transposons carrying kanamycin or chloramphenicol resistance genes [6]–[8]. The finite number of resistance markers has limited genetic analyses to single-gene or single-operon studies, and has prevented complementation of double-deletion strains. As our understanding of *C. jejuni* grows, so does the need for new markers to rapidly delete and restore complex multi-gene systems, and/or to simultaneously express a reporter such as green fluorescent protein (GFP), arylsulfatase, or luciferase in mutant and/or complemented strains. To address this need, we adapted current *C. jejuni* genetic technologies to harbor resistance genes against the antibiotics hygromycin B and apramycin.

Hygromycin B is an aminoglycoside antibiotic produced by *Streptomyces hygroscopicus* that inhibits protein synthesis in both prokaryotes and eukaryotes [9]. Apramycin is another aminoglycoside, an aminocyclitol synthesized by *Streptomyces tenebrarius* [10]. Like other aminoglycosides, such as kanamycin, both hygromycin B and apramycin prevent ribosome translocation during translation elongation by binding the 30 s rRNA proximal to the ribosomal E, P and A sites [11]. Hygromycin B is not used clinically, but is sometimes a component of poultry feed where it has antihelminthic activity against nematode parasites of chickens [12]. Apramycin is also used as a veterinary antibiotic [12], [13]. The hygromycin B (*Hyg^R^*) resistance marker used in this study confers resistance by the activity of a specific **a**minoglycoside **ph**osphotransferase encoded by the 999 bp *aph*(7″) gene, encoding hygromycin B 7″-O-kinase or simply hygromycin phosphotransferase [14]. The specific modification of hygromycin B is a phosphorylation at the 7″-OH of the destomic acid ring [15]. Resistance to apramycin (*Apr^R^*) is conferred by the 777 bp *aac*(3)IV **a**minoglycoside 3-*N*-**ac**etyltransferase gene [13]. Specifically, the enzyme acetylates the 3-amino group of apramycin’s deoxystreptamine ring [16]. Neither *aph*(7″) nor *aac*(3)IV confers resistance to the other’s respective antibiotic, nor do they confer resistance to kanamycin. Vice versa, the *C. jejuni* kanamycin resistance gene *aph*A-3 does not bestow resistance to either hygromycin B or apramycin (data not shown).

In this study, we modified existing *C. jejuni* gene deletion/mutagenesis and insertion strategies and plasmids to encode either *Hyg^R^* or *Apr^R^*. We based our construction of a non-polar mutagenesis construct on the approach devised by Ménard, Sansonetti and Parsot [17], in which the resistance gene is promoterless, does not harbor a terminator, and transcription is driven from the promoter of the operon into which the gene is introduced. We also modified Karlyshev and Wren’s pRRC *C. jejuni* genome-insertional gene delivery and expression system [18], replacing the *cat* **c**hloramphenicol **a**cteyl**t**ransferase cassette with either *aph*(7″) or *aac*(3)IV. The expression of *aph*(7″) and *aac*(3)IV was not detrimental to *C. jejuni* under common laboratory conditions. Furthermore, to demonstrate the potential of these new markers and plasmids, we deleted and then complemented the *C. jejuni* **a**rylsulfate **s**ulfo**t**ransferase *astA,* since the product of *astA* cleaves a chromogenic substance that can be used to report transcriptional activity [19], [20]. With the addition of hygromycin B and apramycin resistance markers, we have provided several new, but relatively familiar, well defined and easy-to-use tools to aid other *Campylobacter* researchers in a variety of genetic approaches.

## Materials and Methods

### Bacterial Strains and Growth Conditions

Bacterial strains and plasmids used in this study are listed in Table 1. *E. coli* strains used for plasmid construction were grown at 37°C in Luria-Bertani (LB, Sigma) broth or on 1.7% (w/v) agar plates supplemented with ampicillin (100 µg mL, Ap), chloramphenicol (15 µg mL^−1^, Cm), kanamycin (50 µg mL^−1^, Kan), hygromycin B (100 µg mL^−1^) or apramycin (50 µg mL^−1^), as necessary. *C. jejuni* strains were grown at 37°C or 42°C in Mueller-Hinton (MH, Oxoid) broth or agar supplemented with vancomycin (10 µg mL^−1^) and trimethoprim (5 µg mL^−1^). *C. jejuni* were grown under standard growth conditions (6% O_2_, 12% CO_2_) using the Oxoid CampyGen system for shaking broth cultures, or in a Sanyo tri-gas incubator for plates. MH was supplemented with chloramphenicol (15 µg mL^−1^), kanamycin (50 µg mL^−1^), hygromycin B (250 µg mL^−1^) or apramycin (60 µg mL^−1^) where appropriate.

**Table 1:** Bacterial strains or plasmids used in this study.

Strain or plasmid	Genotype or description	Source
***E. coli*** ** strains**		
DH5α	F^-^, φ80d *deoR lacZ*Δ*M15 endA1 recA1 hsdR17*(r_K_-m_K_+) *supE44 thi-1 gyrA96 relA1* Δ*(lacZYA-argF) U169*	Invitrogen
***C. jejuni*** ** strains**		
81–176	Wild type isolated from a diarrheic patient	[28]
81–176 pRRH	Strain 81–176 with genome-integrated pRRH; *Hyg^R^*	This study
81–176 pRRA	Strain 81–176 with genome-integrated pRRA; *Apr^R^*	This study
81–176 pRRC	Strain 81–176 with genome-integrated pRRC; *Cm^R^*	This study
81–176 pRRK	Strain 81–176 with genome-integrated pRRK; *Kan^R^*	This study
81–176 Δ*astA::hyg^R^*	Deletion of *astA* with *aph*(7″); *Hyg^R^*	This study
81–176 Δ*astA::apr^R^*	Deletion of *astA* with *aac*(3)IV; *Apr^R^*	This study
DRH461	Strain 81–176 with an unmarked deletion of *astA*	[19]
DRH461 pRRH+*astA*	DRH461 with integrated pRRH and polycistronic promoterless *astA; Hyg^R^*	This study
DRH461 pRRA+*astA*	DRH461 with integrated pRRA and polycistronic promoterless *astA; Apr^R^*	This study
DRH461 pRRH+*astA* (reverse)	DRH461 with integrated pRRH and reverse orientation promoterless *astA; Hyg^R^*	This study
DRH461 pRRA+*astA* (reverse)	DRH461 with integrated pRRA and reverse orientation promoterless *astA; Apr^R^*	This study
DRH461 pRRH+(p)*astA* (reverse)	DRH461 with integrated pRRH and reverse orientation endogenous promoter and *astA; Hyg^R^*	This study
DRH461 pRRA+(p)*astA* (reverse)	DRH461 with integrated pRRA and reverse orientation endogenous promoter and *astA; Apr^R^*	This study
**Plasmids**		
pMV261.hyg	Source of *aph*(7″); *Hyg^R^*	[29], [30]
p261comp.apra	Source of *aac*(3)IV; *Apr^R^*	[30]
pGEM-T	Linearized cloning vector, blue-white screening; *Ap^R^*	Novagen
pBAD24	Low-copy arabinose-inducible expression vector; *Ap^R^*	[31]
pAC1H	pBAD24 ligated to *aph*(7″) amplified with 5631 and 5632; *Hyg^R^, Ap^R^*	This study
pAC1A	pGEM-T ligated to *aac*(3)IV amplified with 5633 and 5634; *Apr^R^, Ap^R^*	This study
pRRC	*C. jejuni* vector for genome integration at rRNA loci; *Cm^R^*	[18]
pRRK	*C. jejuni* vector for genome integration at rRNA loci; *Kan^R^*	J. Ketley
pRRH	*C. jejuni* vector for genome integration at rRNA loci; *Hyg^R^*	This study
pRRA	*C. jejuni* vector for genome integration at rRNA loci; *Apr^R^*	This study
pGEM-T+*astA*	pGEM-T ligated to *astA* amplified with 5707 and 5708; *Ap^R^*	This study
pGEM-T+*astA*::*hyg^R^*	pGEM-T with *astA* interrupted with *aph*(7″) from pAC1H; *Hyg^R^, Ap^R^*	This study
pGEM-T+*astA*::*apr^R^*	pGEM-T with *astA* interrupted with *aac*(3)IV from pAC1A*; Apr^R^, Ap^R^*	This study
pRRH+*astA*	pRRH ligated to *astA* amplified with 0688 and 0689; *Hyg^R^*	This study
pRRA+*astA*	pRRA ligated to *astA* amplified with 0688 and 0689; *Apr^R^*	This study
pRRH+*astA* (reverse)	pRRH ligated to *astA* amplified with 0690 and 0691; *Hyg^R^*	This study
pRRA+*astA* (reverse)	pRRA ligated to *astA* amplified with 0690 and 0691; *Apr^R^*	This study
pRRH+(p)*astA* (reverse)	pRRH ligated to *astA* amplified with 0692 and 0691; *Hyg^R^*	This study
pRRA+(p)*astA* (reverse)	pRRA ligated to *astA* amplified with 0692 and 0691; *Apr^R^*	This study

### Construction of Plasmids pAC1H and pAC1A, pRRH and pRRA

Oligonucleotide primers used in this study are listed in Table 2 and were synthesized by Integrated DNA Technologies. The design of pAC1H and pAC1A plasmids containing the non-polar *aph*(7″) or *aac*(3)IV markers is described in Results. The *aph*(7″) or *aac*(3)IV sequence was amplified from pMV261.hyg or p261comp.apra with ultramer set 5631 and 5632, or 5633 and 5634, respectively. The polymerase chain reaction (PCR) was carried out with iProof high-fidelity DNA polymerase (Bio-Rad). A-ends were incorporated on the purified products by incubation with Taq DNA polymerase (Invitrogen) and dATP. The purified products were then introduced to linearized pGEM-T Easy (Novagen), ligated overnight with T4 DNA ligase (NEB), and transformed into *E. coli* DH5α (Invitrogen). Transformants were selected for on LB media supplemented with ampicillin and either hygromycin B or apramycin.

**Table 2:** Oligonucleotides used in this study (with restriction sites underlined).

Primer	Sequence 5′-3′	Target, sense anddescription	Restrictionsites
5631	ACACCAATTGGGTACCCGGGTGACTAACTAGGAGGAATAA**ATGACACAAGAATCCCTGTTAC**	***aph*** **(7**″**)**, sense, startcodon changed toATG.	*Mfe*I, *Kpn*I, *Sma*I
5632	GTGTGCATGCCTGCAGCATATGTCTAGAGGATCCCCGGGTCATTATTCCCTCCAGGTA**TCAGGCGCCGGGGGCGGTGTCC**	***aph*** **(7**″**)**, antisense.	*Sma*I, *Bam*HI, *Xba*I, *Nde*I, *Pst*I, *Sph*I
5633	ACACCAATTGGGTACCCGGGTGACTAACTAGGAGGAATAA**ATGCAATACGAATGGCGAAAAG**	***aac*** **(3)IV**, sense,start codon changedto ATG.	*Mfe*I, *Kpn*I, *Sma*I
5634	GTGTGCATGCCTGCAGCATATGTCTAGAGGATCCCCGGGTCATTATTCCCTCCAGGTA**TCAGCCAATCGACTGGCGAGCG**	***aac*** **(3)IV**, antisense.	*Sma*I, *Bam*HI, *Xba*I, *Nde*I, *Pst*I, *Sph*I
5705	ACACGGTACCTCCTCCGTAAATTCCGATTTG	pRRC *cat,*antisense.	*Kpn*I
5706	TGGATGAATTACAAGACTTGCTG	pRRC *cat,* sense.	
5707	TATAGGCGAACCAAAAAATCC	Flanking *astA,*sense.	
5708	AAATGTAAATTTGGAAAAGCTTCTC	Flanking *astA,*antisense.	
5709	ACACGGTACCGATCAATCCTTTAAAATTATTTAA	5′-internal *astA,*antisense.	*Kpn*I
5710	ACACTCTAGACAATAAGCCCAAAAATAAATTTGG	3″-internal *astA,*sense.	*Xba*I
0688	ACACTCTAGATAAAGGATTGATCATGAGACTTAG	Promoterless *astA,*sense. Forpolycistronic expression.	*Xba*I
0689	ACACCAATTGATAAGCCCAAATTTATTTTTGGGC	*astA,* antisense.For polycistronicexpression.	*Mfe*I
0690	ACACTCTAGATAAAGGATTGATCATGAGACTTAG	Promoterless *astA,*sense. For reverseexpression.	*Mfe*I
0691	ACACCAATTGATAAGCCCAAATTTATTTTTGGGC	*astA,* antisense.For reverseexpression.	*Xba*I
0692	ACACTCTAGATATAGGCGAACCAAAAAATCC	Upstream *astA,*sense. For reverseexpression.	*Mfe*I

Sequencing verified that the fragment containing *aac*(3)IV was correctly inserted into pGEM-T and this plasmid was then designated pAC1A. Sequencing of pGEM-T containing *aph*(7″) indicated that the restriction sites flanking *aph*(7″) and *aph*(7″) sequence itself were incorrect. The initial *aph*(7″) PCR product was instead digested with *Mfe*I and *Sph*I (NEB), purified, and ligated to low-copy pBAD24 digested with *Eco*RI and *Sph*I. The ligation was transformed into *E. coli* DH5α and sequencing of the transformants indicated the correct *aph*(7″) sequence was incorporated. The resulting plasmid with the *aph*(7″) non-polar marker inserted in pBAD24 was designated pAC1H.

The design of the pRRH and pRRA gene delivery and expression plasmids is also described in the text. Inverse PCR amplification of pRRC was carried out using iProof with primers 5705 and 5706. The resulting PCR product was purified and digested with *Kpn*I and *Xba*I, and ligated to gel-purified *aph*(7″) or *aac*(3)IV markers from similarly-digested pAC1H and pAC1A. Transformants were selected on LB supplemented with hygromycin B or apramycin, and the resulting plasmids were named pRRH or pRRA respectively. *C. jejuni* were transformed with 15 µg of plasmid DNA from pRRH, pRRA, pRRK and pRRC as per established procedure [21] to create antibiotic resistant strains, and verified by PCR against the corresponding resistance gene.

### Growth Analyses and Competition Assays

For standard growth curve analyses, 10 mL overnight broth cultures of *C. jejuni* 81–176 integrated with pRRH (*Hyg^R^*), pRRA (*Apr^R^*), pRRK (*Kan^R^*), and pRRC (*Cm^R^*) were inoculated from growth on agar plates containing the appropriate antibiotic. The next day, at the zero time point, strains were standardized to OD_600_ 0.005 in 10 mL of pre-warmed MH (no antibiotics) and grown for 48 hours shaking at 200 rpm at either 37°C or 42°C. Colony forming units (CFU) were assessed over time by plating 10-fold dilutions of aliquots on MH agar plates. Plates were incubated for 48 hours and colonies counted. To assess relative fitness of each antibiotic resistant strain, a co-culture competition was set up. Cultures were inoculated as above, but at the zero time point, 2.5 mL from each of the 10 mL OD_600_ 0.005 cultures were mixed to create a 10 mL culture containing the 4 marked strains. These were grown at 37°C alongside a wild-type control, and CFU were assessed by plating 10-fold dilutions on MH only, or MH containing one of the four antibiotics. Colonies were counted after 48 hours incubation. Three biological replicates, each with 2 technical replicates, were carried out for each assay.

### Deletion and Complementation of *astA* and Assay for Enzymatic Activity

For mutagenesis, the *astA* gene was PCR-amplified from wild-type 81–176 genomic DNA with primers 5707 and 5708 using iProof DNA polymerase. The PCR product was purified, A-tailed and ligated to pGEM-T to make pGEM-T+*ast*A, which was transformed into *E. coli* DH5α and selected for with ampicillin. Inverse PCR was performed on the resulting plasmid with primers 5709 and 5710 which deleted all 1,863 bp of *astA* and introduced *Kpn*I and *Xba*I sites. The inverse PCR product was digested with *Kpn*I and *Xba*I, ligated to similarly-digested *aph*(7″) or *aac*(3)IV non-polar markers from pAC1H or pAC1A, and transformed into *E. coli* DH5α. The resulting constructs, pGEM-T+*astA::hyg^R^* and pGEM-T+*astA::apr^R^*, were purified, verified and then transformed into *C. jejuni* 81–176 to create Δ*astA::hyg^R^* and Δ*astA::apr^R^*. For complementation, iProof PCR was used to amplify *astA* with primer sets 0688 and 0689 (promoterless *astA*), 0690 and 0691 (promoterless *astA* in reverse), and 0691 and 0692 (promoter and *astA* in reverse). This introduced *Xba*I and *Mfe*I restriction sites to each of the 3 products. The PCR products, and pRRH and pRRA, were digested with *Xba*I and *Mfe*I, and the plasmids were dephosphorylated with Antarctic Phosphatase (NEB). Following clean-up, each *astA* gene was ligated to each plasmid and transformed into DH5α. Colonies were screened by PCR for inserts, sequenced, and the resulting plasmids were introduced into a Δ*astA* strain, DRH461 [19]. To assess arylsulfatase activity, overnight cultures of bacteria were standardized to OD_600_ 0.05 and 10 µL of bacterial culture was spotted on MH agar supplemented with 100 µg mL^−1^ of 5-bromo-4-chloro-3-indolyl sulfate potassium salt (XS, Sigma). For quantification, the liquid arylsulfatase assay was carried out as described [19], [21], with the exception that strains were incubated in AB3 buffer for 2 h instead of 1 h. Two biological replicates, each with two technical replicates, were carried out.

## Results

### Creation of Hygromycin and Apramycin Resistance Markers for *C. jejuni* Gene Replacement/Deletion

**Figure 1:**
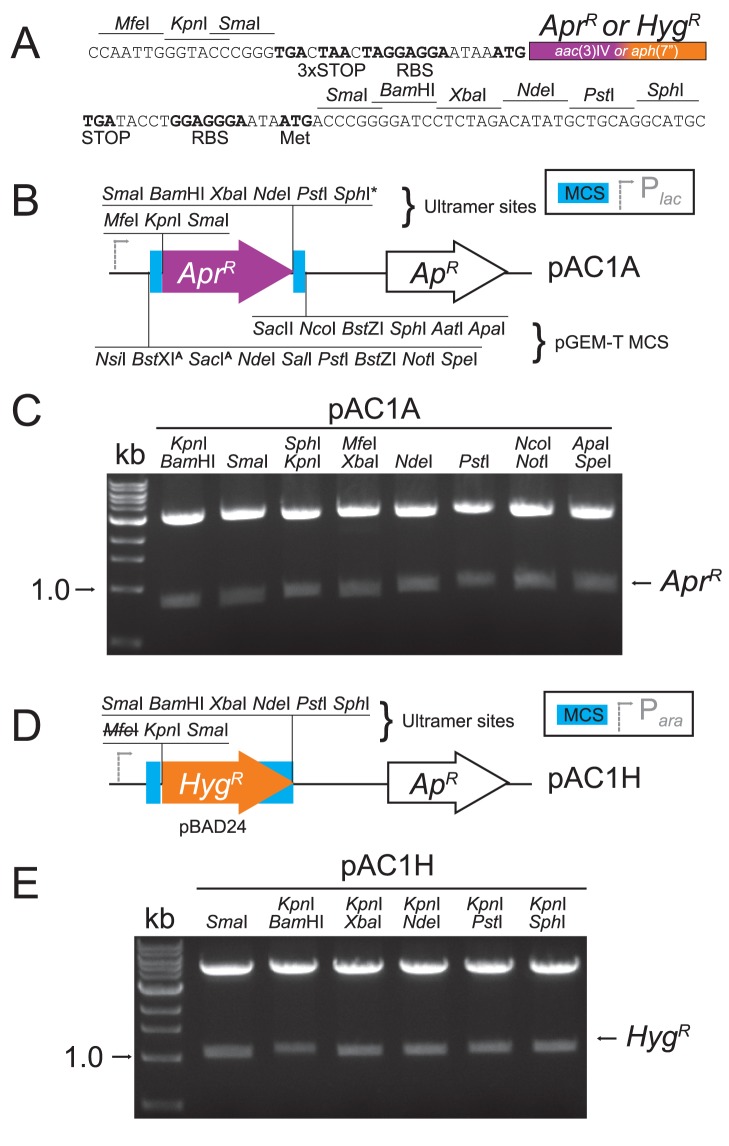
Synthesis of plasmids containing *aph*(7″) or *aac*(3)IV as non-polar hygromycin B and apramycin resistance markers. (A) Schematic of ultramers designed to amplify *aph*(7″) or *aac*(3)IV. The 5′ ultramers 5631 and 5633, for *aph*(7″) or *aac*(3)IV respectively, include *Mfe*I, *Kpn*I and *Sma*I restriction sites, stop codons in all three reading frames, and a ribosome binding site. The 3′ ultramers 5632 and 5634 include a ribosome binding site, a start codon in-frame with restriction sites for *Sma*I and *Bam*HI, and restriction sites for *Xba*I, *Nde*I, *Pst*I and *Sph*I. (B) The amplified *aac*(3)IV was introduced by TA cloning into linearized pGEM-T, conserving the restriction sites in the pGEM-T multiple cloning site (MCS). The resulting plasmid is pAC1A. The pGEM sites may also be used for the sub-cloning of the apramycin resistance marker (*Apr^R^*). MCS sites that cut *aac*(3)IV are indicated with a superscript ‘A’. (C) All introduced sites in pAC1A were tested by restriction digest. (D) The *Mfe*I- and *Sph*I-digested *aph*(7″) amplification product was cloned into pBAD24 digested with *Eco*RI (*Mfe*I-compatible) and *Sph*I. The *Mfe*I site was lost in the resulting plasmid, pAC1H. (E) All restriction sites introduced to pAC1H were tested by digest.

To construct *Hyg^R^* and *Apr^R^* cassettes that could be used for mutagenesis, we synthesized PCR ultramers to *aph*(7″) or *aac*(3)IV, which included the restriction enzyme cut sites and features depicted in Fig. 1A based on the non-polar *Kan^R^* cassette described by Ménard, Sansonetti and Parsot [17]. This construct contains neither a promoter nor a transcription terminator, with the resistance genes preceded at the 5′-end by translational stop codons in all reading frames and also including a Shine-Dalgarno sequence or ribosome binding site (RBS). The 3′-end is followed by another RBS, multiple restriction sites for cloning, and a start codon upstream of and in-frame with the *Sma*I and *Bam*HI cut sites. This start codon is designed to overcome translational coupling of genes in polycistrons if the *Sma*I or *Bam*HI cut sites are employed. The ***A*** *pr^R^* construct was subsequently introduced into high-copy pGEM for clonal amplification (pAC1**A**, Fig. 1B). However, unwanted recombination and loss of restriction cut sites flanking the ***H*** *yg^R^* gene necessitated introducing the *Hyg^R^* construct into the low-copy pBAD24 vector instead (pAC1**H**, Fig. 1D). Via restriction analyses, we confirmed that all introduced restriction sites can be effectively used to excise the resistance cassettes (Fig. 1 C, E). When harbored by *E. coli*, expression of the resistance markers is driven by *lac* or *ara* inducible promoters in pGEM and pBAD respectively; however, induction was not required for *E. coli* growth in the presence of the corresponding antibiotic. Each antibiotic resistance cassette, lacking a transcriptional terminator, is thus now in an *E. coli* cloning vector convenient for non-polar insertional mutagenesis and constructing additional clones for *C. jejuni* manipulation.

### Modification of the pRRC Genome-insertional Gene Delivery Vector to Carry *aph*(7″) or *aac*(3)IV

**Figure 2:**
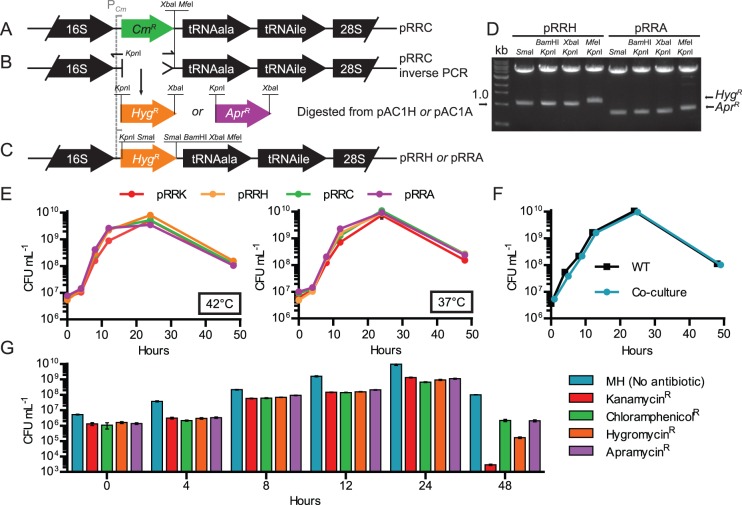
Adaptation of the pRRC gene delivery and expression system to harbor hygromycin B or apramycin resistance, and testing of genome-integrated markers for detrimental effects of resistance genes. (A) Schematic of pRRC, which inserts into any of 3 rRNA clusters in the genome by homologous recombination. (B) Inverse PCR amplification of pRRC with primers 5705 (*Kpn*I) and 5706 deleted the chloramphenicol resistance gene but conserved the *Campylobacter*-optimized *cat* promoter. (C) The inverse PCR product was digested with *Kpn*I and *Xba*I, and ligated to similarly digested *aph*(7″) or *aac*(3)IV from pAC1H or pAC1A to create pRRH and pRRA respectively (only pRRH is shown). (D) Restriction digest analysis confirmed the function of all introduced sites. (E) The resistance markers from pRRK, pRRC, pRRH and pRRA were inserted into the *C. jejuni* 81–176 genome, and each resulting strain was analyzed for microaerobic growth and survival in shaken Mueller-Hinton (MH) broth by counting CFU over 48 hours at both 42°C (left panel) and 37°C (right panel). (F) To determine if the introduction of either marker contributed any fitness cost that could affect competitiveness against wild-type or the other marked strains, a competition assay was performed. Equal numbers of wild-type marked with hygromycin, apramycin, chloramphenicol and kanamycin resistance markers were co-cultured with unmarked wild-type in shaking MH broth at 37°C under microaerobic conditions. CFU were assessed by plating a dilution series on MH agar. (G) CFU were further assessed from the co-culture by plating on MH only (the total CFU, same data as in F) or MH supplemented with each antibiotic, representing the number of bacteria resistant to each antibiotic.

The chloramphenicol resistance marker (*cat*, *Cm^R^*) encoded on vector pRRC (Fig. 2A) [18] was exchanged with *Hyg^R^* or *Apr^R^*. The endogenous *aph*(7″) or *aac*(3)IV promoters were non-functional in *C. jejuni* (data not shown), so the pRRC *cat* promoter was retained to ensure expression. Exchange of *cat* was achieved using an inverse PCR methodology, in which a *Kpn*I site was introduced to the 5′-end of the antisense primer (Fig. 2B). The antisense primer was targeted to the DNA immediately upstream of *cat*, allowing conservation of the *cat* promoter. The inverse PCR product was next digested with *Kpn*I and *Xba*I and ligated to the marker from a similarly-digested pAC1H or pAC1A. For the insertion of genes, the resulting plasmids, pRR**H** and pRR**A** (Fig. 2C), for ***H*** *yg^R^* or ***A*** *pr^R^* respectively, now include an additional *Bam*HI site in addition to the *Xba*I and *Mfe*I sites present in pRRC. Both pRRA and pRRH also contain *Sma*I sites that flank the resistance cassette; as such, these *Sma*I sites cannot be used for gene insertion for complementation or heterologous gene expression purposes. Functionality of all sites was confirmed by restriction enzyme mapping (Fig. 2D).

### The Presence of *aph*(7″) or *aac*(3)IV in *C. jejuni* is not Detrimental to Growth

To test if the hygromycin B or apramycin resistance genes affected *C. jejuni* growth and survival, pRRH and pRRA were integrated into the genome of *C. jejuni* 81–176 to create *Hyg^R^* or *Apr^R^* wild-type strains. In the absence of their respective antibiotics, we assessed the time-course growth profile of these strains and compared CFU recovered to those of wild-type and wild-type marked with *aph*A-3 from pRRK and *cat* from pRRC. Neither the *Hyg^R^* or *Apr^R^* strains were defective for growth in MH broth under microaerobic conditions in at 37°C or 42°C, the optimal temperature range of the bacterium (Fig. 2E). However, because differences in fitness cost of the antibiotic markers may not have been detected in the first experiment, we also carried out a competitive index-style assay. Broth cultures were inoculated with equal numbers of each of the 4 resistant strains, and the mixed cultures were grown at 37°C alongside a wild-type only control (Fig. 2F). At each time point, CFU were assessed by plating dilutions on MH or MH containing hygromycin B, apramycin, kanamycin or chloramphenicol. The total CFU were represented on the MH-only plate, while the number of resistant bacteria were determined on each antibiotic-containing plate. No fitness cost was observed for either *Hyg^R^* or *Apr^R^* strains between 0–24 hours (Fig. 2G). At 48 hours there was a modest decrease in CFU recovered for *Hyg^R^* strains; however, this was less pronounced than the defect observed for strains carrying the well-established *Kan^R^* marker. It should be noted that at the 48 hour timepoint, each culture exhibited an overall decrease in the relative CFU recovered under antibiotic selection, suggesting that older cultures are generally more sensitive to antibiotic pressure.

### Deletion and Complementation of Arylsulfatase *astA* with *Hyg^R^* or *Apr^R^* Constructs

**Figure 3:**
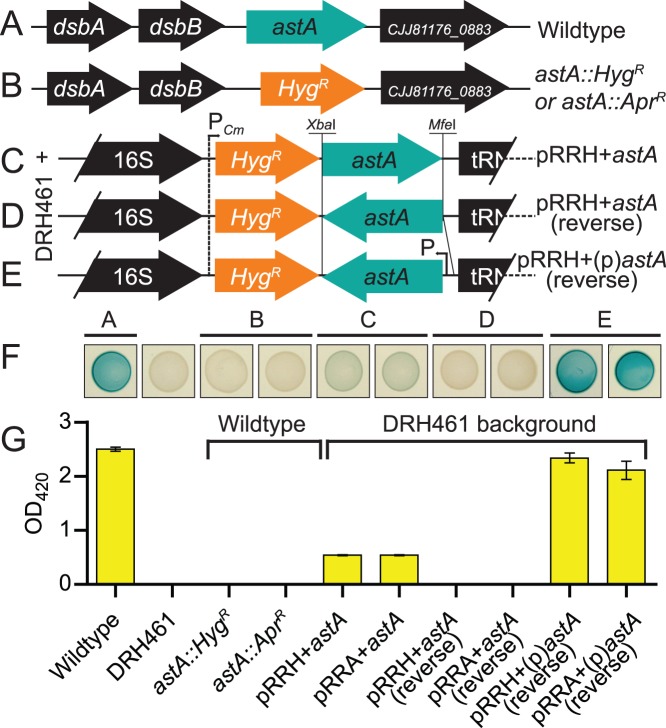
Mutagenesis of the arylsulfatase gene *astA* with *aph*(7″) or *aac*(3)IV non-polar markers and complementation of Δ*astA* via genomic insertion with pRRH or pRRA. (A) Loci arrangement of *astA* single-gene operon in *C. jejuni* 81–176. (B) Deletion of *astA* with either *aph*(7″) or *aac*(3)IV from pAC1H or pAC1H. (C) Introduction of promoterless *astA* into pRRH or pRRA in the same orientation as the *cat* promoter created pRRH+*astA* or pRRA+*astA* and resulted in polycistronic expression of *astA* with *aph*(7″) or *aac*(3)IV. (D) Promoterless *astA* inserted in the opposite orientation to the *cat* promoter (designated pRRH+*astA* (reverse) or pRRA+*astA* (reverse) (E) Insertion of the endogenous *astA* promoter and *astA* in the opposite orientation to the *cat* promoter in pRRH and pRRA created pRRH+(p)*astA* (reverse) and pRRA+(p)*astA* (reverse). Only *Hyg^R^* plasmids/strains are depicted in B–E, but both *Hyg^R^* and *Apr^R^* plasmids represented with *Hyg^R^* in C, D and E were integrated into the genome of the Δ*astA* strain, DRH461. (F) Arylsulfatase activity of the deletion and complementation strains was assessed by spotting 10 µL of OD-standardized cultures onto MH agar plates supplemented with the chromogen XS cleaved by arylsulfatase. A blue-green color indicates activity, and the spots correspond to labels on the bar graph below. (G) Quantification of arylsulfatase activity from broth cultures to assess transcription of *astA*.

In *C. jejuni*, expression of arylsulfatase (*astA*) can be monitored via colorimetric plate and broth assays [19], [21]. To further test the usability of pAC1H, pAC1A, pRRH and pRRA, we mutagenized *astA* with the non-polar *Hyg^R^* or *Apr^R^* markers from pAC1H and pAC1A and also restored a copy of *astA* to the genome of a Δ*astA* strain using pRRH or pRRA. First, *astA* (Fig. 3A) was completely deleted and replaced with the non-polar *Hyg^R^* or *Apr^R^* markers from pAC1H and pAC1A, respectively (Fig. 3B). Next, a promoterless *astA* was cloned into pRRH or pRRA in the transcriptional direction of, and thus expressed by, the *cat* promoter (Fig. 3C), and these constructs were integrated into DRH461, an unmarked Δ*astA* strain [19]. The *astA* gene was also cloned without (Fig. 3D) and with (Fig. 3E) its native promoter into pRRH or pRRA in the reverse orientation to the *cat* promoter. These latter constructs test expression only from the endogenous *astA* promoter and were likewise integrated into DRH461. Each strain was spotted onto MH solid media containing the chromogenic substrate XS, which is cleaved by arylsulfatase [20], and grown for 72 hours. No arylsulfatase activity was observed in any deletion strain. Partial complementation was observed for *astA* expressed from the *cat* promoter, full complementation was observed for *astA* expressed from its native promoter, and no complementation was observed for the promoterless *astA* cloned in the reverse orientation to the *cat* promoter (Fig. 3F). A quantitative liquid spectrophotometric assay confirmed the plate readouts (Fig. 3G).

## Discussion

With the introduction of hygromycin B and apramycin resistance markers, we have provided researchers in the *C. jejuni* field with additional genetic tools, essentially doubling the number of broadly usable markers for this organism. We envision that these markers will be especially useful for deletion of multiple genes, complementation, and/or the addition of various promoter-reporter constructs. Although it is possible to construct a potentially unlimited number of unmarked deletion mutants in *C. jejuni* [7], a disadvantage of unmarked mutations is that they cannot be easily transferred from one genetic background to another. This is especially critical for an organism like *C. jejuni* with high rates of phenotypic variation associated with phase-variable, highly mutable contingency loci [1], [22], [23]. This variation is often unpredictable and may result in phenotypes unlinked to the intended mutation, and it is often pertinent to test several mutant clones or re-introduce a marked mutation to a wild-type background to ensure the veracity of any phenotype.

The non-polar markers harbored by gene disruption cassettes in pAC1H and pAC1A have both advantages and disadvantages when compared to markers that also introduce a promoter, such as the *C. jejuni cat* cassette in pRY109 [5]. Primarily, the main advantage of a promoterless disruption construct is that the introduction of a non-endogenous promoter affects transcription of any co-transcribed gene at the 3′-end of the deleted gene in an operon. Vice versa, one disadvantage of the promoterless marker is that it is reliant on transcription from the promoter of the gene into which it has inserted. Therefore, if the target gene is not highly expressed, then the same will be true for the resistance gene, and antibiotic resistance will not be conferred to the bacterium. Our laboratory has experienced this problem, and if a mutant cannot be made with a non-polar marker, then it can be attempted using the *cat* cassette from pRY109. Another advantageous feature of the non-polar markers in pAC1H and pAC1A is the start codon at the 3′-end of the resistance gene; specifically, use of the 3′ *Sma*I or *Bam*HI restriction sites allows placement of the start codon in the reading frame of the stop codon of the mutated gene. This configuration allows for translation of a remainder of the gene into which the marker is inserted, preventing polar effects on the downstream gene due to translational coupling. Translational coupling is the interdependence of translation efficiency of co-transcribed genes on a polycistronic mRNA [17], [24]. We do not routinely design our deletion/replacement strategies to take translational coupling into account for the initial study of a gene; however, translational coupling was recently observed for the *C. jejuni dsb* genes [25], so remains an important consideration.

Many of the features of the pRRC vector were conserved in the new pRRA and pRRH gene delivery vectors. In particular, the *cat* promoter is common to all three plasmids, which is advantageous because it allows for constitutive expression of an introduced gene as part of the polycistronic mRNA coding the resistance marker. This is helpful for the expression of genes with no directly adjacent promoters, such as those found within operons. However, as we demonstrated in Fig. 3 F, G, the level of polycistronic expression can be out of context with that of the endogenous system. For *astA*, expression from the *cat* promoter is considerably lower than from the *astA* promoter. Likewise, for a gene that is not highly expressed, or expressed only under certain conditions, expression from the *cat* promoter can be higher than normal or expressed at inappropriate times. Nonetheless, under some circumstances the *cat* promoter can provide full complementation, as we and others have shown [18], [26], [27]. A new advantage of pRRA and pRRH is the addition of the *Bam*HI site in addition to the pre-existing *Xba*I and *Mfe*I sites, which permits greater choice in the directional cloning of insertions for complementation and heterologous expression.

The fitness cost of harboring *aph*(7″) or *aac*(3)IV was minimal or comparable to either kanamycin or chloramphenicol resistance markers (Fig. 2G). In addition, the length of time required for transformants to grow on media containing either hygromycin B or apramycin was comparable to that of kanamycin-supplemented MH. Transformants also appeared 3–4 days earlier than with chloramphenicol, reducing the time needed to delete or introduce a gene. For cloning in *E. coli*, 50–100 µg mL^−1^ of apramycin or 100–250 µg mL^−1^ hygromycin B was generally sufficient. In *C. jejuni*, we found that 60 µg mL^−1^ of apramycin was the optimal concentration for selection. The minimum inhibitory concentration of hygromycin B for *C. jejuni* was determined to be >32 µg mL^−1^ (not shown), but this value was not useful in determining a working concentration, possibly because the high culture densities typically used in *C. jejuni* transformation protocols easily overcame the antibiotic. By trial-and-error, we found that 250 µg mL^−1^ hygromycin B was necessary to ensure selection. Apramycin and hygromycin B are also attractive reagents because they are relatively inexpensive drugs, although the high concentrations of hygromycin B increase the cost of using *aph*(7″) as a marker. One potential drawback of *aac*(3)IV is that it is reported to confer resistance to gentamicin [16], an antibiotic used to kill bacteria during assessments of host cell invasion. However, our *Apr^R^* (*aac*(3)IV) strains behaved identically to other marked strains in gentamicin-protection assays, indicating that invasion and intracellular survival can be accurately assessed with mutants harboring this marker (data not shown).

In summary, we have adapted a set of well-established plasmids to encode hygromycin B and apramycin resistance for gene deletion, replacement, and expression in *C. jejuni*. We also established the optimal concentrations of both hygromycin B and apramycin for the purposes of selection. In addition, we determined that introduction of *aph*(7″) or *aac*(3)IV was not detrimental and that there was no appreciable fitness cost to *C. jejuni* when compared to chloramphenicol or kanamycin resistance markers. These constructs were validated using the *astA* reporter, and are currently being utilized in our laboratory for exploratory studies of uncharacterized genes. These new molecular tools will provide a broader range of possible experiments, will assist in the mechanistic study of *C. jejuni*, and contribute to a better understanding of the microorganism’s lifecycle and pathogenicity.
